# Association of serum gamma-glutamyltransferase and premature coronary artery disease

**DOI:** 10.1007/s12471-017-0964-5

**Published:** 2017-02-15

**Authors:** M. Sheikh, M. Tajdini, A. Shafiee, M. Sotoudeh Anvari, A. Jalali, H. Poorhosseini, A. Amirzadegan

**Affiliations:** 1 Tehran Heart Center, Tehran University of Medical Sciences, Tehran, Iran; 2 Department of Community Medicine, Faculty of Medicine, Shahid Beheshti university of Medical sciences, Tehran, Iran

**Keywords:** Acute coronary syndrome, Premature coronary artery disease, Coronary angiography, Gamma-glutamyltransferase

## Abstract

**Background:**

Serum gamma-glutamyltransferase (GGT) has been introduced as a predictive factor for cardiovascular disease. In this study, we investigated the association of serum GGT and premature coronary artery disease (CAD) in candidates for coronary angiography.

**Methods:**

In this cross-sectional study, we enrolled male subjects aged ≤45 years and female subjects ≤55 years who were candidates for elective coronary angiography due to typical chest pain or a positive non-invasive test. Baseline characteristics were recorded for all the participants and serum levels of blood glucose, lipid profile and GGT were measured. Patients were divided into CAD and non-CAD groups based on angiography for further comparisons.

**Results:**

From a total of 367 patients (age 45.1 ± 6.1 years, 161 males [43.9%]), 176 (47.9%) patients had premature CAD. A high level of GGT was significantly associated with the presence of CAD (*p* < 0.001). A 10-unit increase in GGT could strongly predict the presence of premature coronary artery disease (OR: 13.34, 95% CI: 7.19–24.78; *p* < 0.001) after adjustment for confounders. The area under the receiver operating characteristic curve for GGT was 80.9% (range 76.5–85.3) and the sensitivity and specificity of GGT at a cut-point of 22.5 IU/l was 80.1% and 70.2%, respectively. Diagnostic accuracy of GGT was 74.9%. The positive predictive value and negative predictive value for GGT was 71.3 and 79.3, respectively.

**Conclusion:**

We observed that GGT levels in patients with typical chest pain or positive non-invasive tests could predict the presence of premature CAD in young patients.

## Introduction

Coronary artery disease (CAD) is a complex multifactorial condition that is initiated by an intense inflammatory response to different forms of injury, leading to endothelial dysfunction in the arterial walls and finally resulting in accelerated atherosclerosis [1]. Premature CAD, defined as the presence of coronary artery atherosclerotic lesion in men ≤45 years and women ≤55 years [2] is a growing entity in developing countries such as Iran. At least one of the four classic cardiovascular risk factors – cigarette smoking, diabetes, hypertension, and dyslipidaemia – is present in the majority (almost 90%) of patients with premature CAD [3]. Some of the cardiovascular risk factors, such as family history, smoking, hypertension, and diabetes mellitus [2, 4], have shown to be strong predictors for premature CAD, while others were not [5]. As coronary angiography remains the gold standard for the diagnosis of CAD [6], identification of patients with premature CAD remains a challenge until they refer with cardiovascular events. Therefore, earlier prediction of this serious condition can help to reduce cardiovascular mortality and morbidity. Finding tools to identify individuals at risk and identification of novel predictive factors, including biomarkers, could greatly help to detect this condition in its early phases, [2].

Elevation of serum gamma-glutamyltransferase (GGT), an important liver enzyme, mediates the preparation of amino acid to intracellular glutathione and also has a role in the degradation of extracellular glutathione [7]. Being the most important non-protein cellular antioxidant, glutathione degradation has a pro-oxidant role in various conditions, such as low-density lipoprotein oxidation [8]. These provide evidence for the pathological impact of a rise in GGT on stimulating oxidative processes and on regulating the antioxidant defence system. Accordingly, it can be postulated that GGT may play a role in the inflammatory process underlying atherosclerosis. Previous studies have demonstrated the association of serum GGT with cardiovascular disease, diabetes, and metabolic syndrome [9–11]; it has been suggested as a predictive factor for the incidence of CAD and cardiovascular-related mortality [12, 13]. However, there are insufficient data regarding the association of GGT and premature CAD. The aim of this study was to investigate such an association in patients undergoing coronary angiography at our tertiary heart centre.

## Methods

### Study population

In this cross-sectional study, between March 2012 and September 2013 we enrolled male subjects aged ≤45 years and female subjects ≤55 years who were referred to Tehran Heart Center for elective coronary angiography due to typical chest pain or positive non-invasive test results (i. e. electrocardiogram suggestive of ischaemia, positive exercise tolerance test, or suspicious myocardial perfusion scan). Exclusion criteria were malignancy, metabolic syndrome, abnormal liver function tests (increased level of GGT combined with elevated aspartate aminotransferase, alanine aminotransferase, and bilirubin is due to liver dysfunction and possibly biliary tract involvement, so these patients were excluded), active hepatic disease, and chronic renal/hepatic insufficiency. Patients with a history of known CAD or myocardial infarction, cardiac surgery, and regular alcohol consumption (more than 90 ml daily for at least 6 months), were excluded from the study.

This study conforms to the principles outlined in the Declaration of Helsinki. Written informed consent was obtained from the participants before enrolment. The study protocol was approved by the local Institutional Committee of Medical Ethics and the Research Board of Tehran University of Medical Sciences.

### Demographic and clinical data

Baseline characteristics, including demographic data and past medical history, were recorded for all the participants in a face-to-face interview at the time of admission. A positive history of hypertension was considered in patients already taking antihypertensive agents or who had two blood pressure readings at least five minutes apart in the sitting posture ≥140/90 mm Hg. Dyslipidaemia was considered in patients taking lipid-lowering agents, total cholesterol ≥200 mg/dl, or low density lipoprotein ≥130 mg/dl. Diabetes mellitus was positive in patients with a definite history of diabetes and treatment with glucose-lowering agents or a fasting plasma glucose ≥126 mg/dl or two-hour post-load glucose ≥200 mg/dl.

### Laboratory measurements

Venous blood samples were obtained before coronary angiography from the antecubital vein at the catheterisation laboratory of our centre to measure serum biochemistry. These measurements included fasting blood glucose, lipid profile (i. e. triglyceride, total cholesterol, low-density lipoprotein [LDL], and high-density lipoprotein [HDL]), liver function tests (aspartate aminotransferase, alanine aminotransferase, and bilirubin) and serum GGT. Serum GGT was measured using Cobas Integra 400 plus (Roche Diagnostics, Basel, Switzerland) by enzymatic colorimetric assay.

### Angiography

The presence of premature CAD was evaluated by conventional angiography at the catheterisation laboratory of Tehran Heart Center under local anaesthesia by an expert cardiologist. The angiography was performed by the standard Judkins technique using a quantitative coronary angiographic system. Significant atherosclerotic coronary artery lesion was defined according to the guideline of the American College of Cardiology/American Heart Association as a 50% or more narrowing of the lumen diameter in at least one major coronary artery [14].

All coronary angiograms were evaluated by a qualified cardiologist unaware of the patient’s biochemical markers results. The extent of coronary atherosclerosis was assessed with the clinical vessel score and CAD was defined as the presence of ≥50% stenosis in the coronary arteries: single-vessel disease indicated stenosis in the left anterior descending artery or left circumflex artery or right coronary artery or a main branch of one of these arteries; double-vessel disease is stenosis in two coronary arteries other than the left main artery, and triple-vessel disease refers to stenosis in three coronary arteries [15]. The study variables were then compared between the groups with and without significant CAD. Also, the relationship between serum GGT and the extent of coronary artery disease based on the vessel score was examined.

### Statistical analysis

Continuous variables were described with mean and standard deviation (SD) or with median and 25th and 75th percentiles for skewed data, and were compared between CAD and non-CAD groups using the Student’s t or Mann-Whitney U test where appropriate. Categorical variables were expressed as frequency and percentage and were compared among groups using the chi-square test. The area under the receiver operating characteristic (ROC) curve with 95% confidence interval (CI) was applied to estimate the discrimination power of cardiovascular risk factors and GGT. Variables which were simultaneously associated with CAD and GGT with *p*-values less than 0.2 were considered potential confounders. A multiple logistic regression model was applied to evaluate the association of GGT and CAD adjusted for detected potential confounders. The adjusted effect was reported through odds ratio (OR) with 95% CI. A Kruskal-Wallis test was applied to compare GGT among groups of disease severity. Pairwise comparisons were used to find different groups in GGT. *P*-values less than 0.05 were considered statistically significant. The statistical analysis was performed using IBM SPSS statistics for windows version 22.0 (Armonk, NY: IBM Corp.).

## Results

The mean age of the study participants was 45.1 ± 6.1 years and 43.9% were men. Based on the coronary angiography results, from a total of 367 patients 176 (47.9%) had various degrees of CAD. The frequency of diabetes mellitus, hypertension and smoking were significantly higher in patients with premature CAD (*p* = 0.001, *p* = 0.02 and *p* = 0.025, respectively). Levels of fasting blood glucose as well as GGT were significantly higher in the CAD group (*p* = 0.024 and *p* < 0.001, respectively). Only one patient reported one episode of alcohol consumption within the past 6 months. Comparison of the study variables between the CAD and non-CAD groups is summarised in Table 1.

**Table 1:** Comparing the baseline characteristics of the study groups based on the presence of premature coronary artery disease

Parameters^a^	CAD+ (*n* = 176)	CAD− (*n* = 191)	*P*-value*
Age, year	45.6 ± 5.6	45.0 ± 6.8	0.53
Male gender, *n* (%)	85 (48.3)	76 (39.8)	0.1
Diabetes mellitus, *n* (%)	65 (36.9)	41 (21.5)	0.001
Hypertension, *n* (%)	88 (50)	73 (38.2)	0.02
Dyslipidaemia, *n* (%)	85 (48.3)	96 (50.3)	0.70
Smoking, *n* (%)	57 (32.4)	42 (22)	0.02
Family history of CAD, *n* (%)	57 (32.4)	56 (29.3)	0.52
Fasting blood sugar, mg/dl	126.9 ± 56.0	110.8 ± 40.2	0.02
Triglyceride, mg/dl	178.8 ± 104.2	168.2 ± 92.3	0.3
Total cholesterol, mg/dl	184.5 ± 58.3	176.3 ± 47.5	0.13
LDL, mg/dl	119.8 ± 45.2	112.4 ± 36.6	0.08
HDL, mg/dl	39.2 ± 10.2	44.5 ± 10.5	<0.001
GGT, IU/l	43.3 ± 12.1	21.46 ± 12.8	<0.001

*CAD* coronary artery disease, *GGT* gamma-glutamyl transferase, *HDL* high density lipoprotein cholesterol, *LDL* low-density lipoprotein cholesterol

*p < 0.05 was considered statistically significant

^a^Variables are shown as mean ± standard deviation or frequency (percentage) where appropriate

In the multivariable logistic regression analysis, after adjustment for gender, diabetes mellitus, hypertension, smoking, fasting blood glucose, cholesterol, triglyceride and HDL, a 10-unit elevation in serum GGT was significantly associated with a twofold increase in the risk of premature CAD (OR: 2.03, 95% CI: 1.70–2.41.78; *p* < 0.001).

The area under the ROC curve (AUC), sensitivity, and specificity of cardiovascular risk factors for detecting premature CAD in our study population is presented in Table 2. Serum GGT had the largest AUC for discriminating premature CAD followed by low HDL and high fasting blood glucose. Based on the logistic regression model, analysis was performed for Framingham risk factors (including age, total cholesterol, HDL, smoking and hypertension) and significant premature CAD; findings revealed an AUC of 0.69 for males and 0.71 for females (*p* < 0.001). Analysis was repeated after adding GGT to these factors and findings showed the AUC for males to be 0.81 and for females 0.86 (*p* < 0.001). Based on Table 3 the AUC for GGT alone is 0.77 for males and 83.2 for females (*p* < 0.001). So, although adding GGT to the Framingham risk factors increases their value, GGT has a more important role on its own (Table 3).

**Table 2:** Receiver operating characteristic (ROC) analysis of cardiovascular risk factor components for discriminating premature coronary artery disease from normal coronary structure

Risk factor	AUC	95% CI	Sensitivity	Specificity	*P*-value*
Diabetes mellitus	57.7	51.9–63.6	36.9	78.5	0.01
Hypertension	55.9	50.0–61.8	50	61.8	0.05
Smoking	55.2	49.3–61.1	32.4	78	0.08
FBS (cut-point 102.5 mg/dl)	58.40	52.6–64.3	53.40	66.50	0.05
LDL (cut-point 144 mg/dl)	53.4	47.4–59.3	27.8	83.8	0.265
HDL (cut-point 45.5 mg/dl)	64.70	59.1–70.3	45.5	72.70	<0.001
GGT (cut-point 22.5 IU/l)	80.90	76.5–85.3	80.10	70.20	<0.001

*AUC* area under the ROC curve, *FBS* fasting blood sugar, *GGT* gamma-glutamyltransferase, *HDL* high-density lipoprotein cholesterol

*p < 0.05 was considered statistically significant

**Table 3:** Diagnostic test indices of Framingham risk factors with and without GGT for predicting premature coronary artery disease in the study population and men and women separately

Population	AUC		*P*-value*
	Males	Females	
Framingham risk factors without GGT	0.69	0.71	<0.001
Framingham risk factors with GGT	0.81	0.86	<0.001

Framingham risk factors: age, total cholesterol, HDL, hypertension, smoking

*AUC* area under the ROC curve, *GGT* gamma-glutamyltransferase

*p < 0.05 was considered statistically significant

**Fig. 1:**
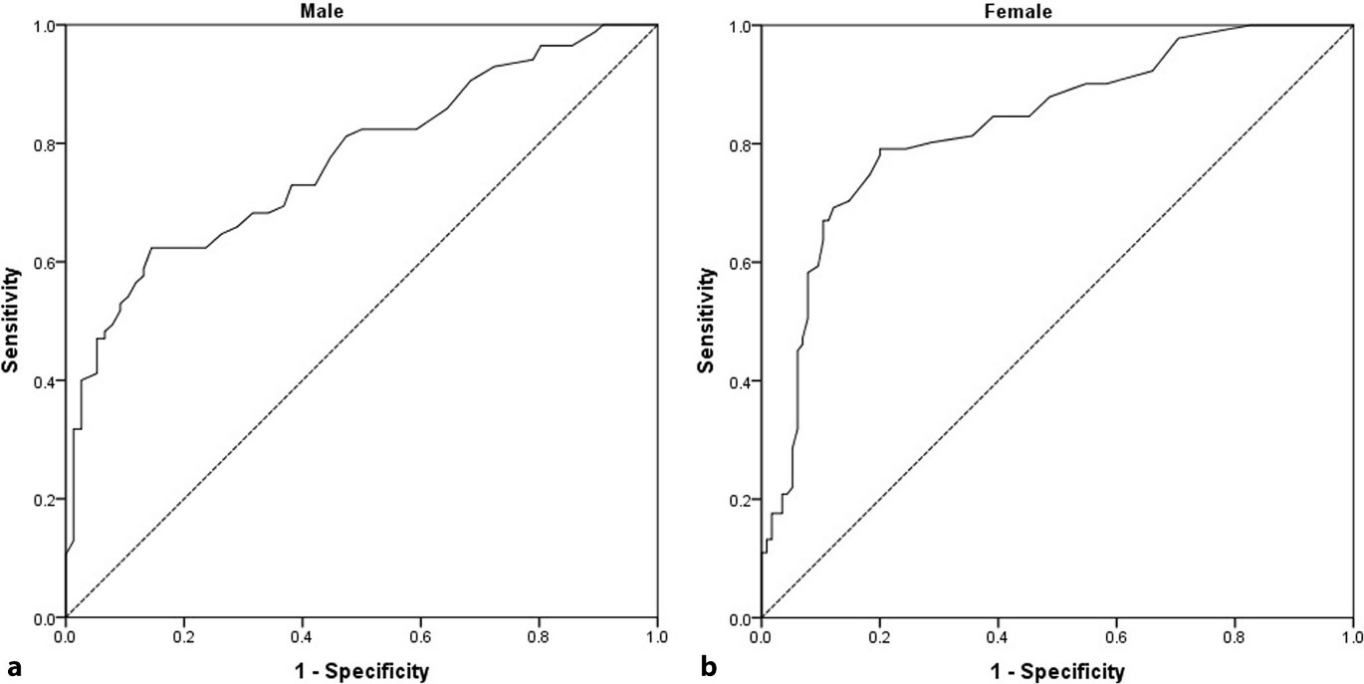
The receiver operating characteristic curve for serum GGT used for screening of high-risk CAD patients. **a** Males (AUC = 77.1%, *p* < 0.001), **b** Females (AUC = 83.2%, *p* < 0.001)

As shown in Fig. 1, the positive and negative predictive values for GGT were 71.2 (95% CI: 64.4–77.4) and 79.3 (95% CI: 72.4–85.1), respectively. Diagnostic test indices of GGT as well as cut-off points for predicting premature CAD in the study population plus men and women separately are shown in Table 4.

**Table 4:** Diagnostic test indices of GGT for predicting premature coronary artery disease in the study population and men and women separately

Population	GGT cut-off	AUC	95% CI	Sensitivity	Specificity	PPV	NPV	Diagnostic accuracy	*P*-value*
All patients	22.5	80.9	76.5–85.3	80.1	70.2	71.3	79.3	74.9	<0.001
Men	35.5	77.1	76.5–85.3	62.4	86.5	82.1	67	73.3	<0.001
Women	22.5	83.2	77.0–88.2	79.1	80	75.8	82.9	79.6	<0.001

*AUC* area under the ROC curve, *CI* confidence interval, *GGT* gamma-glutamyltransferase, *NPV* negative predictive value, *PPV* positive predictive value

*p < 0.05 was considered statistically significant

**Fig. 2:**
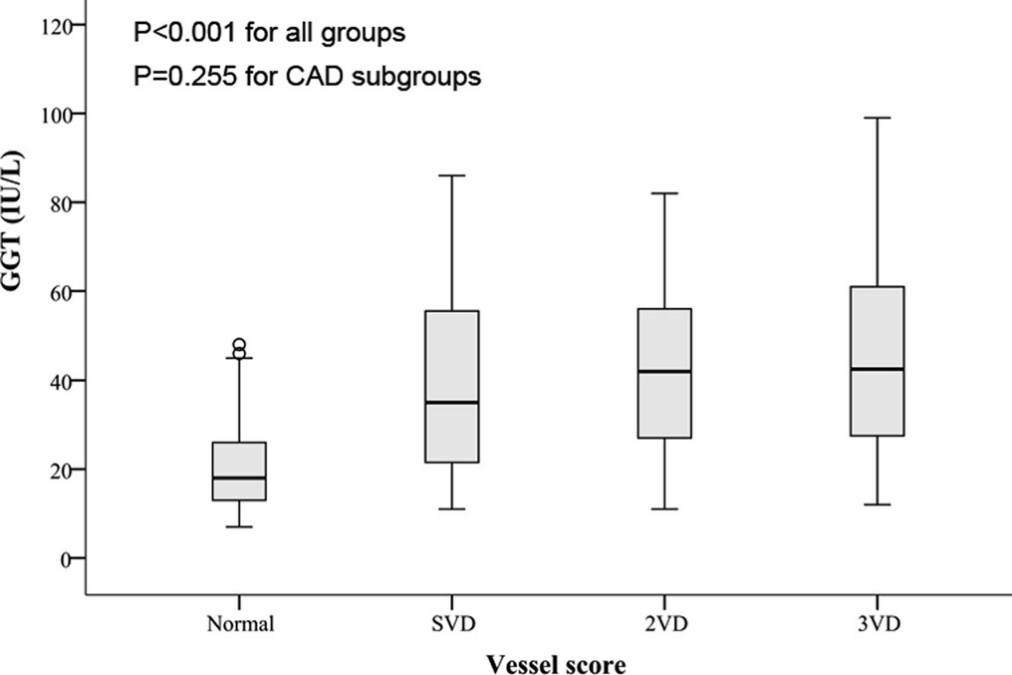
Box plot of GGT levels in the subgroup of patients based on the presence and extent of premature CAD as defined by vessel score (single vessel disease [*SVD*] is defined as stenosis [at least 50%] of LAD, LCX, RCA or a main branch of one of them, two-vessel disease [*2VD*] is stenosis of two coronary arteries other than the left main, and triple-vessel disease [*3VD*] is stenosis in three coronary arteries)

The levels of GGT were also compared between different subgroups of vessel score and we observed that the level of GGT was significantly lower in normal coronary individuals (*p* < 0.001) (Fig. 2). However, the post-hoc pairwise comparison revealed that this difference was caused by low concentrations of GGT in normal coronary patients and patients with one, two, or three vessel disease did not statistically differ regarding the GGT levels (*p* = 0.255).

## Discussion

The main finding of the present study was that GGT levels in the studied patients could predict the presence of premature CAD with an acceptable precision. We also observed that GTT was significantly associated with the extent of premature CAD as measured by the angiographic vessel score.

Oxidative stress and the resultant endothelial dysfunction contribute to the micro-vascular complications of metabolic disease and atherosclerosis [16, 17]. Common risk factors for atherosclerosis stimulate production of free oxygen radicals, either by endothelial cells or by vascular smooth muscle cells and adventitial cells [18]. Thus, dyslipidaemia, diabetes mellitus, hypertension, smoking, advanced age, and nitrate intolerance all play a part in overproduction of reactive oxygen species [19, 20]. As a result, several processes involved in atherogenesis will be initiated, including expression of adhesion molecule, stimulation of vascular smooth muscle proliferation and migration, endothelial apoptosis, lipid oxidation, matrix metalloproteinase activation, and alteration in vasomotor activity [21, 22]. Already a biomarker for hepatobiliary disease and alcohol abuse [7], serum GGT activity has been introduced as a biomarker for oxidative stress with an apparent association with increased risk of hypertension, diabetes mellitus, and cardiovascular events [23, 24]. It also intermediates LDL oxidation through glutathione/GGT-dependent iron reduction process as a part of atherosclerosis pathogenesis [8]. Moreover, GGT has been shown to be a valid predictor for cardiovascular mortality and survival in patients with cardiovascular disease. Therefore, one can conclude that GGT has tight bonds with cardiovascular conditions, particularly in early stages of coronary atherosclerosis [13].

As coronary angiography is an invasive test for the detection of CAD, GGT can play an invaluable role in predicting the presence of premature CAD in the young adults who present with ischaemic symptoms. Presence of the classic cardiovascular risk factors can predict the presence of CAD to some degree; however, addition of GGT can increase the predictive strength. The comparison between the predictors of CAD in the present study showed that GGT can predict the presence of premature CAD with a high precision as compared with other risk factors. This finding is in line with the result of a similar study that also suggested GGT as an adjuvant marker for risk assessment of premature CAD and a screening tool for the candidates of coronary angiography [25]. The cut-point for GGT in the abovementioned study is similar to the cut-point for the male patients in our study as the number of female patients in that study was very low. Therefore, we suggest the use of serum GGT measurement as a non-invasive tool for predicting premature CAD in the young adults who present with ischaemic symptoms.

### Study limitations

The advantage of our study is that we provided the cut-points of GGT for both men and women. However, the main limitation of this study is its design; this was a single-centre study performed in a tertiary university hospital and this may influence our findings due to demographic and socioeconomic reasons.

## Conclusion

Our results showed that GGT, as a cost-effective and quick test, could predict the presence of premature CAD with a diagnostic accuracy of 74.9% in our patients. Although further studies are required to find out the definite cut-off values for GGT in different populations, the association of GGT with premature CAD suggests that it can be used as an adjuvant marker for risk assessment of young patients who present with ischaemic symptoms and are suspected to have premature CAD.
